# Impact of Residual Mediastinal Nodal Disease After Neoadjuvant Chemoimmunotherapy for Non-Small Cell Lung Cancer

**DOI:** 10.3390/cancers18172871

**Published:** 2026-09-05

**Authors:** Berta Mosleh, Anastasia Papaporfyriou, Thorsten Fuereder, Helmut Prosch, Joachim Widder, Felicitas Oberndorfer, Clemens Aigner, Marco Idzko, Daniela Gompelmann, Mir Alireza Hoda

**Affiliations:** 1 Department of Thoracic Surgery, Medical University of Vienna, 1090 Vienna, Austria; berta.mosleh@meduniwien.ac.at (B.M.); clemens.aigner@meduniwien.ac.at (C.A.); 2 Comprehensive Cancer Center Vienna, Medical University of Vienna, 1090 Vienna, Austria; anastasia.papaporfyriou@meduniwien.ac.at (A.P.); thorsten.fuereder@meduniwien.ac.at (T.F.); helmut.prosch@meduniwien.ac.at (H.P.); joachim.widder@meduniwien.ac.at (J.W.); felicitas.oberndorfer@meduniwien.ac.at (F.O.); marco.idzko@meduniwien.ac.at (M.I.); daniela.gompelmann@meduniwien.ac.at (D.G.); 3 Comprehensive Center for Chest Diseases, Medical University of Vienna, 1090 Vienna, Austria; 4 Division of Pulmonology, Department of Internal Medicine II, Medical University of Vienna, 1090 Vienna, Austria; 5 Division of Oncology, Department of Medicine I, Medical University of Vienna, 1090 Vienna, Austria; 6 Department of Biomedical Imaging and Image-Guided Therapy, Medical University of Vienna, 1090 Vienna, Austria; 7 Department of Radiation Oncology, Medical University of Vienna, 1090 Vienna, Austria; 8 Department of Pathology, Medical University of Vienna, 1090 Vienna, Austria

**Keywords:** non-small cell lung cancer, neoadjuvant chemoimmunotherapy, residual mediastinal nodal disease, nodal staging, disease-free survival

## Abstract

**Background/Objectives:** In recent years, neoadjuvant chemoimmunotherapy followed by surgery has improved outcomes in patients with stage II/III non-small cell lung cancer (NSCLC). However, the prognostic impact of residual mediastinal nodal disease after chemoimmunotherapy remains insufficiently defined, particularly with the introduction of the Union for International Cancer Control/American Joint Committee on Cancer (UICC/AJCC) 9th edition nodal subclassification. **Methods:** In this retrospective study, 90 consecutive patients with clinically node-positive (cN1-2) resectable locally advanced NSCLC who underwent neoadjuvant chemoimmunotherapy followed by curative-intent resection were included. Disease-free survival (DFS) was analyzed according to postoperative pathologic nodal status. Nodal status was assessed using the UICC/AJCC 8th edition (ypN0, ypN1, ypN2) and reclassified according to the 9th edition (ypN0, ypN1, ypN2a, ypN2b). Residual postoperative nodal disease was defined as residual pathologic nodal involvement (ypN+) in patients with clinically node-positive (cN+) disease at baseline. **Results:** The cohort comprised 90 patients (60% male, median age 63 years [interquartile range (IQR) 58–69]). Following neoadjuvant therapy, surgery revealed pathologic nodal clearance in 53 (58.9%) and residual nodal disease in 37 (41.1%) patients (ypN1, *n* = 16 [17.8%]; ypN2a, *n* = 15 [16.7%]; and ypN2b, *n* = 6 [6.7%]). DFS differed significantly by postoperative nodal status (global log-rank *p* < 0.001), with progressively poorer outcomes with increasing residual nodal burden. **Conclusions:** Residual nodal disease after neoadjuvant chemoimmunotherapy and surgery is associated with progressively inferior DFS according to its anatomic extent. The UICC/AJCC 9th-edition staging system provides clinically relevant prognostic granularity, supporting further investigation of mediastinal restaging and postoperative treatment strategies.

## Simple Summary

Residual nodal disease after neoadjuvant chemoimmunotherapy may identify a high-risk group among patients with resectable, node-positive non-small cell lung cancer, but its prognostic relevance and anatomic extent remain insufficiently characterized. Previous studies have largely relied on a binary postoperative classification of complete nodal clearance versus persistent nodal disease. In this consecutive cohort of 90 patients treated with neoadjuvant chemoimmunotherapy followed by curative resection, disease-free survival progressively worsened with increasing residual nodal burden. Application of the UICC/AJCC 9th-edition nodal classification further refined postoperative stratification by distinguishing single-station from multi-station disease, yielding a clear survival gradient across ypN0, ypN1, ypN2a, and ypN2b categories. Residual mediastinal disease remained independently associated with inferior disease-free survival after adjustment for pathologic response of the primary tumor. These findings identify the anatomic extent of residual nodal disease as a clinically relevant determinant of postoperative prognosis and support prospective evaluation of mediastinal restaging and adjuvant treatment strategies.

## 1. Introduction

Worldwide, lung cancer remains the leading cause of cancer-related mortality despite substantial advances in screening and treatment. For resectable disease, anatomic resection with mediastinal lymphadenectomy remains the cornerstone. However, recurrence after surgery alone occurs in up to 55% of patients [1,2], while neoadjuvant or adjuvant chemotherapy provides only a modest 5–15% improvement in 5-year overall survival [3,4,5,6]. The advent of immunotherapy has opened a new era in lung cancer treatment. Immune checkpoint inhibitors have transformed the management of advanced non-small cell lung cancer (NSCLC), yielding durable survival benefits in metastatic and locally advanced unresectable disease. These successes have catalyzed interest in integrating immunotherapy at earlier stages, including neoadjuvant and perioperative settings [7].

In resectable NSCLC, neoadjuvant immunotherapy combined with platinum-based chemotherapy has rapidly changed the treatment landscape. While neoadjuvant chemoimmunotherapy has become a cornerstone of curative-intent management in stage III disease, emerging data now support its use in stage II NSCLC [8]. Phase II and randomized phase III trials have demonstrated that neoadjuvant chemoimmunotherapy significantly increases rates of major pathologic response (MPR) and pathologic complete response (pCR) with improved event-free and overall survival compared with chemotherapy alone [9,10,11,12,13]. In CheckMate 816, neoadjuvant nivolumab plus chemotherapy increased the pCR rate to 24.0% versus 2.2% with chemotherapy alone and prolonged median event-free survival from 20.8 to 31.6 months (HR 0.63) [9]. Similarly, the perioperative KEYNOTE-671 and AEGEAN trials demonstrated significant event-free survival benefits with pembrolizumab (HR 0.58) and durvalumab (HR 0.68), respectively, together with substantially higher pCR rates than chemotherapy alone [10,11]. However, prospective trials have predominantly reported overall ypN downstaging and pathologic response rates without stratifying residual hilar or mediastinal disease versus complete nodal clearance after neoadjuvant chemoimmunotherapy and surgery [9,14,15]. Consequently, the survival implications of persistent N1/N2 disease remain unclear [16,17]. In the pre-immunotherapy era, persistent mediastinal nodal disease following induction chemotherapy or chemoradiotherapy was consistently associated with inferior survival compared with nodal clearance [18,19,20,21]. While retrospective analyses in the immunotherapy era show that MPR and pCR correlate with improved outcomes, the prognostic impact of residual nodal disease is less clearly characterized [15,22,23]. Notably, these studies classified nodal disease according to the 8th edition of the Union for International Cancer Control/American Joint Committee on Cancer (UICC/AJCC) staging system and did not distinguish single- from multi-station mediastinal disease, as introduced in the revised 9th edition, implemented after January 2025 [24]. At present, the prognostic significance of these new N2 subcategories after neoadjuvant chemoimmunotherapy and surgery remains undefined.

## 2. Materials and Methods

### 2.1. Study Design

This retrospective, single-center cohort study included consecutive patients with histologically confirmed, baseline clinically node-positive (cN+) stage II–III NSCLC who underwent neoadjuvant chemoimmunotherapy followed by curative-intent surgery at the Department of Thoracic Surgery, Medical University of Vienna, Austria, between 1 January 2017, and 30 June 2025. All patients underwent molecular profiling before treatment initiation. Patients with targetable oncogenic driver alterations were excluded.

This study was conducted in accordance with the Declaration of Helsinki and approved by the Ethics Committee of the Medical University of Vienna, Austria (EK Nr: 1753/2025, date of approval: 1 October 2025). Owing to the retrospective nature of the study, informed consent was waived.

### 2.2. Study Objectives and Endpoints

The primary objective was to evaluate disease-free survival (DFS) according to postoperative pathologic nodal status following neoadjuvant chemoimmunotherapy and resection. Postoperative nodal status was evaluated according to the UICC/AJCC 8th edition (ypN0, ypN1, ypN2) and the 9th edition (ypN0, ypN1, ypN2a, ypN2b). The primary endpoint was DFS, defined as the interval from surgery to disease recurrence or death from any cause. Secondary objectives were to evaluate the association between pathologic response and DFS, to assess integrated risk stratification based on pathologic response and residual nodal status, and to identify clinical and pathologic factors associated with residual nodal disease.

### 2.3. Patient Population

Patients with stage II–III NSCLC deemed resectable by qualified thoracic surgeons at multidisciplinary thoracic oncology tumor boards were eligible. Patients received neoadjuvant treatment in line with randomized clinical trial evidence. Neoadjuvant treatment comprised platinum-based chemotherapy combined with an immune checkpoint inhibitor, including pembrolizumab (56.7%), nivolumab (42.2%), or durvalumab (1.1%), typically administered for 3–4 cycles. Postoperative treatment was protocol-defined before treatment initiation: pembrolizumab- and durvalumab-based regimens represented perioperative treatment strategies including adjuvant immunotherapy, whereas nivolumab-based regimens were neoadjuvant-only. In accordance with the consensus recommendations from the International Association for the Study of Lung Cancer [25], response to neoadjuvant therapy was assessed using contrast-enhanced chest CT and/or PET/CT, followed by multidisciplinary reassessment. Invasive mediastinal restaging was reserved for selected patients with equivocal findings, accounting for 7.8% of our study population. Patients without radiologic progression or persistent bulky mediastinal disease, while remaining medically operable and technically resectable according to our institutional protocols [26] and the contemporary EORTC recommendations [27], proceeded to surgery.

Curative-intent anatomic lung resection was performed by thoracotomy or video-assisted thoracoscopic surgery (VATS), combined with systematic mediastinal and hilar/intrapulmonary lymphadenectomy. Complete (R0) resection was achieved in all patients. Lymphadenectomy included systematic dissection of at least three mediastinal (N2) lymph node stations, including the subcarinal station, and at least one hilar or intrapulmonary (N1) station, according to institutional protocols aligned with established surgical guidelines.

### 2.4. Data Collection

Clinical, radiologic, pathologic, and oncologic data were extracted retrospectively from medical records. Variables included demographics, smoking history, tumor histology and localization, clinical TNM stage at diagnosis, PD-L1 tumor proportion score, treatment regimen and timing, and type of resection. Postoperative nodal status and distribution were derived from surgical specimens, classified according to the UICC/AJCC 8th edition (ypN0, ypN1, ypN2) and reclassified using the 9th edition nodal subcategories (ypN2a and ypN2b). Pathologic response was categorized as pCR, MPR, or no objective pathologic response according to established histopathological criteria [28]. All surgical specimens were evaluated by experienced thoracic pathologists. In resected lymph nodes containing residual tumor cells, extranodal extension was routinely assessed during institutional histopathologic evaluation, documented in the final pathology reports when present, and subsequently reviewed by multidisciplinary tumor boards.

### 2.5. Risk Stratification Based on Pathologic Response and Residual Nodal Disease

Patients were classified by pathologic response and postoperative nodal status, two established determinants of outcome after neoadjuvant therapy and resection. The low-risk group comprised pCR/MPR with complete nodal clearance (ypN0); the intermediate-risk group included pCR/MPR with ypN1 or no objective response with ypN0; the high-risk group included all patients with ypN2 or no objective response with ypN1.

### 2.6. Statistical Analysis

Categorical variables are presented as frequencies and percentages, and continuous variables as medians and interquartile ranges (IQR). Groups were compared using the χ^2^ test or Fisher’s exact test for categorical variables and the Mann–Whitney U test for continuous variables. DFS was defined as the time from surgery to recurrence or death; event-free patients were censored at last follow-up. DFS was analyzed according to pathologic nodal status (UICC/AJCC 8th and 9th editions), pathologic response, and integrated risk categories. DFS was estimated using the Kaplan–Meier method and compared using the log-rank test. Hazard ratios (HRs) with 95% confidence intervals (CIs) were estimated using Cox proportional hazards regression. Given the limited number of DFS events, the multivariable survival analysis was restricted to a two-variable Cox model including residual mediastinal nodal disease (ypN2 vs. ypN0/ypN1) and objective pathologic response (pCR/MPR vs. no objective response). The exploratory comparison of ypN2b versus ypN2a disease was performed using a univariable Cox model. In the integrated risk analysis, risk category was modeled as an ordinal variable. Median follow-up was calculated using the reverse Kaplan–Meier method. Univariable logistic regression analyses were performed to explore factors associated with residual mediastinal nodal disease, defined as ypN2 versus ypN0/ypN1. Because only 21 patients had residual ypN2 disease, the multivariable model was restricted to baseline clinical nodal status (cN2 vs. cN1) and pretreatment PD-L1 expression to reduce overfitting. PD-L1 was modeled continuously and reported per 10-percentage-point increase. Logistic-regression results are reported as odds ratios (ORs) with 95% CIs. Analyses were performed using complete-case data. A two-sided *p*-value < 0.05 was considered statistically significant. The sample size was determined by the number of consecutive eligible patients available during the predefined retrospective study period; therefore, no prospective sample size calculation was performed. Analyses were performed using SPSS Statistics version 30.0 (IBM Corp., Armonk, NY, USA). Graphics were generated using GraphPad Prism version 8.4.3 (GraphPad Software, Boston, MA, USA).

## 3. Results

### 3.1. Patients and Demographics

The study cohort comprised 90 patients, including 54 men (60.0%) and 36 women (40.0%), with a median age of 63 years (IQR 58–69). Histologically, 52 patients (57.8%) had adenocarcinoma and 38 (42.2%) had squamous cell carcinoma. Clinical stage distribution according to the UICC/AJCC 8th edition was stage IIB in 8 patients (8.9%), stage IIIA in 32 patients (35.6%), and stage IIIB in 50 patients (55.6%). Baseline clinical nodal status was cN1 in 24 patients (26.7%) and cN2 in 66 patients (73.3%).

Tumors were located in the right lung in 50 (55.6%) and the left lung in 38 (42.2%) patients; two (2.2%) were centrally located at the level of the carina. Pembrolizumab-based treatment corresponding to the KEYNOTE-671 perioperative strategy was administered in 51 patients (56.7%), nivolumab-based treatment corresponding to the CheckMate 816 neoadjuvant-only strategy in 38 patients (42.2%), and durvalumab-based treatment corresponding to the AEGEAN perioperative strategy in 1 patient (1.1%). The median number of neoadjuvant treatment cycles was 3 (IQR 3–4). Serious adverse events during neoadjuvant therapy occurred in 9 patients (10.0%), including pancytopenia in 3 patients (3.3%), pneumonitis in 2 (2.2%), neutropenia in 2 (2.2%), arthritis in 1 (1.1%), and hypophysitis with Addisonian crisis in 1 (1.1%). None of these events precluded subsequent curative-intent surgery.

The median interval between completion of neoadjuvant treatment and surgery was 5.6 weeks (IQR 4.0–6.7). Resection was performed via open thoracotomy in 72 patients (80.0%) and via video-assisted thoracoscopic surgery (VATS) in 18 patients (20.0%). Lobectomy was the most frequently performed procedure (69/90, 76.7%), followed by pneumonectomy (13/90, 14.4%) and bilobectomy (6/90, 6.7%). Two patients (2.2%) underwent carinal resection. Lobar sleeve resections were performed in 11 patients (12.2%). Patients requiring pneumonectomy represented a highly selected subgroup with locally advanced and centrally located tumors for which an R0 parenchyma-sparing resection was not technically and oncologically feasible. Ten of thirteen patients (76.9%) had baseline cT3-4 disease. In contrast, sleeve lobectomy was successfully performed in 11 patients (12.2%) when adequate oncologic margins could be achieved without pneumonectomy. Post-treatment hilar or nodal fibrosis/adherence increased the complexity of vascular dissection in two patients, both of whom required pulmonary arterioplasty/vascular sleeve resection, but did not necessitate extension of the pulmonary resection. Postoperative complications occurred in 15 patients (16.7%). Prolonged air leak was the most frequent complication (*n* = 7, 7.8%), followed by hemothorax, recurrent laryngeal nerve dysfunction/hoarseness, chylothorax, and atrial fibrillation (*n* = 2 each, 2.2%). Baseline characteristics are summarized in Table 1.

### 3.2. Residual Nodal Disease

Nodal involvement was histologically confirmed in 80 patients (88.9%) by endobronchial ultrasound-guided transbronchial needle aspiration (EBUS-TBNA). In the remaining 10 patients (11.1%), clinical nodal status was determined radiologically based on PET/CT imaging, following multidisciplinary tumor board evaluation. After neoadjuvant chemoimmunotherapy and resection, 53 of 90 patients (58.9%) achieved nodal clearance (ypN0), whereas 37 (41.1%) exhibited residual nodal disease (ypN1 or ypN2). Residual ypN1 disease was observed in 16 patients (17.8%). According to the UICC/AJCC 8th edition, residual mediastinal disease (ypN2) was present in 21 patients (23.3%). Using the UICC/AJCC 9th edition, mediastinal disease was further subclassified into ypN2a (single-station involvement) in 15 patients (16.7%) and ypN2b (multi-station involvement) in 6 patients (6.7%). No extranodal extension (extracapsular nodal spread) was identified in the resected lymph nodes.

### 3.3. Nodal Yield and Extent of Lymphadenectomy

The median number of resected lymph nodes was 11 (IQR 8–17), including a median of 7 N2 nodes (IQR 4–10) and 4 N1 nodes (IQR 2–7.8). The higher numerical N2 yield should be interpreted in the context of the predominantly cN2 study population and systematic multi-station mediastinal lymphadenectomy. In clinically involved mediastinal stations, enlarged or confluent nodal tissue may result in several individually identifiable lymph nodes. Patients with residual nodal disease had a median of 14 resected nodes (IQR 8–20) versus 11 (IQR 8–15) in those achieving ypN0 (*p* = 0.140). Among patients with residual nodal disease (*n* = 37), the median number of positive lymph nodes was 2 (IQR 1–3). The median lymph node ratio (LNR, defined as the number of pathologically positive lymph nodes divided by the total number of resected lymph nodes) was 0.17 (IQR 0.10–0.33), with values of 0.13 in ypN1 and 0.20 in ypN2 disease (*p* = 0.066). Postoperative nodal status is summarized in Table 2.

### 3.4. Disease-Free Survival by Residual Nodal Disease

**Figure 1:**
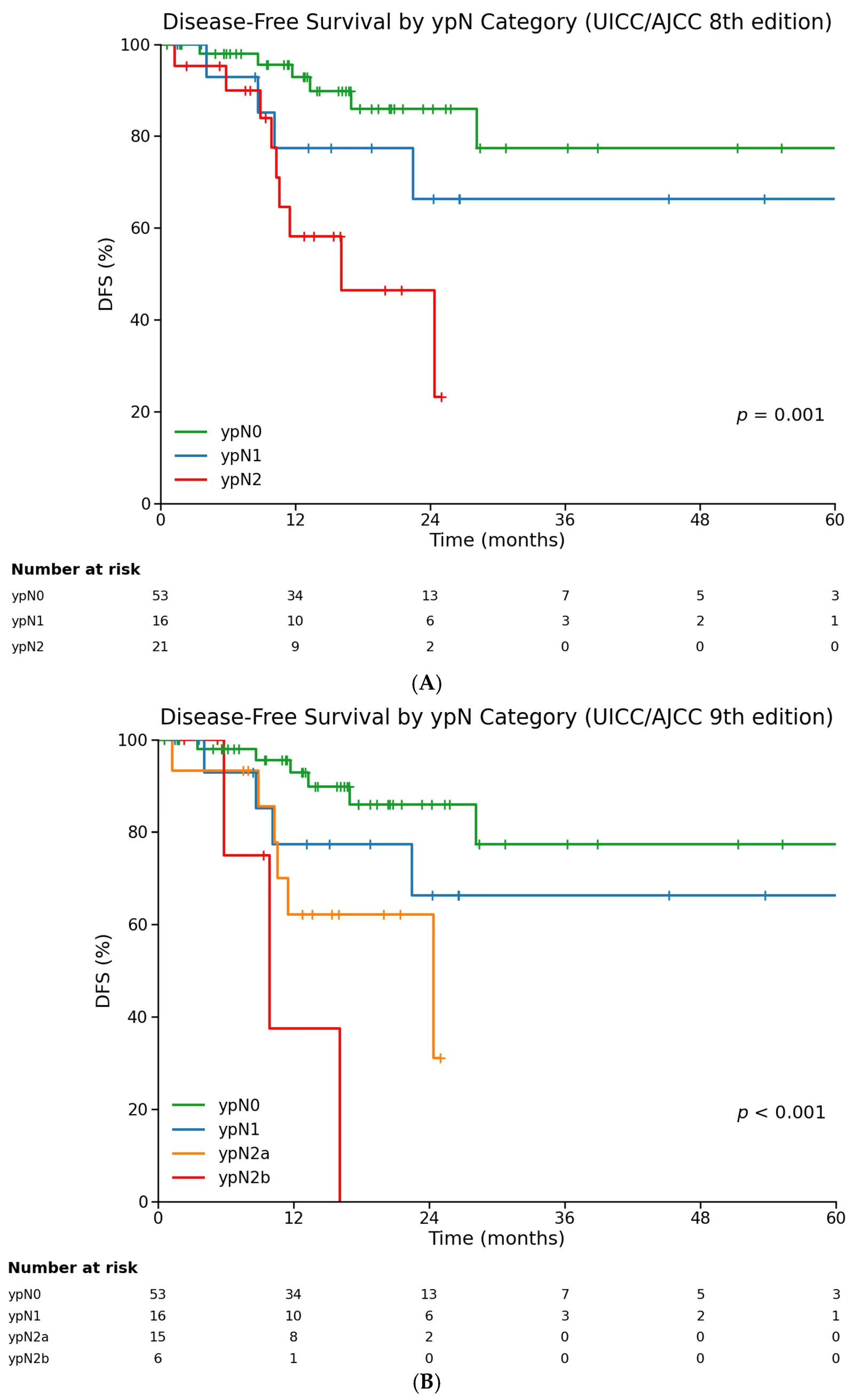
Disease-free survival by postoperative nodal status (ypN) according to the 8th and 9th editions of the UICC/AJCC staging systems. Kaplan–Meier curves representing DFS stratified by postoperative nodal status following neoadjuvant treatment and resection in patients with clinically node-positive disease (cN+). (**A**) DFS according to the ypN classification (ypN0, ypN1, ypN2) based on the UICC/AJCC 8th edition demonstrated a significant stepwise decrease with increasing residual nodal disease (global log-rank *p* = 0.001). Median DFS was not reached for ypN0 disease, was 64.8 months (95% CI, 10.2–64.8) for ypN1 disease, and 16.1 months (95% CI, 10.3–not reached) for ypN2 disease. (**B**) DFS according to the ypN classification based on the UICC/AJCC 9th edition (ypN0, ypN1, ypN2a, ypN2b) further refined prognostic heterogeneity across postoperative nodal categories (global log-rank *p* < 0.001). Median DFS was not reached for ypN0 disease, was 64.8 months (95% CI, 10.2–64.8) for ypN1 disease, 24.4 months (95% CI, 10.3–not reached) for ypN2a disease, and 9.9 months (95% CI, 5.8–16.1) for ypN2b disease. Numbers at risk are shown below each Kaplan–Meier curve.

Median follow-up was 16.9 months. During follow-up, 20 DFS events occurred, including 18 recurrences and 2 deaths without documented recurrence. Seventy patients were censored without an event. DFS differed significantly by postoperative nodal status following neoadjuvant therapy. Using the UICC/AJCC 8th edition (Figure 1A), DFS declined stepwise with increasing residual nodal disease (ypN0, ypN1, ypN2; global log-rank *p* = 0.001). Median DFS was not reached in the ypN0 group, was 64.8 months (95% CI, 10.2–64.8) in those with ypN1 disease, and was markedly shorter at 16.1 months (95% CI, 10.3–not reached) in those with ypN2 disease. The ypN1 median estimate should be interpreted cautiously because few patients remained at risk during late follow-up. The UICC/AJCC 9th edition (Figure 1B) further refined prognostic heterogeneity across postoperative nodal categories (global log-rank *p* < 0.001). Patients with ypN2a and ypN2b disease demonstrated distinct outcomes, with median DFS values of 24.4 months (95% CI, 10.3–not reached) and 9.9 months (95% CI, 5.8–16.1), respectively; these subgroup estimates were considered exploratory because of the small subgroup sizes.

In an exploratory direct comparison limited to patients with residual mediastinal nodal disease, ypN2b was associated with numerically worse DFS than ypN2a (HR 3.15, 95% CI, 0.72–13.77; direct log-rank *p* = 0.109), although the small number of ypN2b patients precluded definitive conclusions. In an exploratory parsimonious Cox proportional hazards model including residual mediastinal nodal disease (ypN2 vs. ypN0/ypN1) and objective pathologic response (pCR/MPR vs. no objective response), residual mediastinal nodal disease remained associated with inferior DFS (HR 3.33, 95% CI 1.21–9.11; *p* = 0.019), whereas pathologic response was not independently associated with DFS (HR 2.15, 95% CI 0.76–6.06; *p* = 0.148).

Postoperative treatment was protocol-driven and independent of postoperative pathologic findings. Overall, 52 patients were treated according to perioperative immunotherapy protocols, including adjuvant treatment, and 38 according to neoadjuvant-only protocols. Perioperative/adjuvant immunotherapy protocols were used in 28 of 53 ypN0 patients, 9 of 16 ypN1 patients, 9 of 15 ypN2a patients, and 6 of 6 ypN2b patients. Among patients with residual nodal disease (ypN1–2), DFS did not significantly differ between patients who received adjuvant treatment and those who did not (median DFS, 22.5 months [95% CI, 10.6 months–not reached] vs. 24.4 months [95% CI, 5.8–64.8 months], respectively; *p* = 0.810).

### 3.5. Disease-Free Survival by Pathologic Response

**Figure 2:**
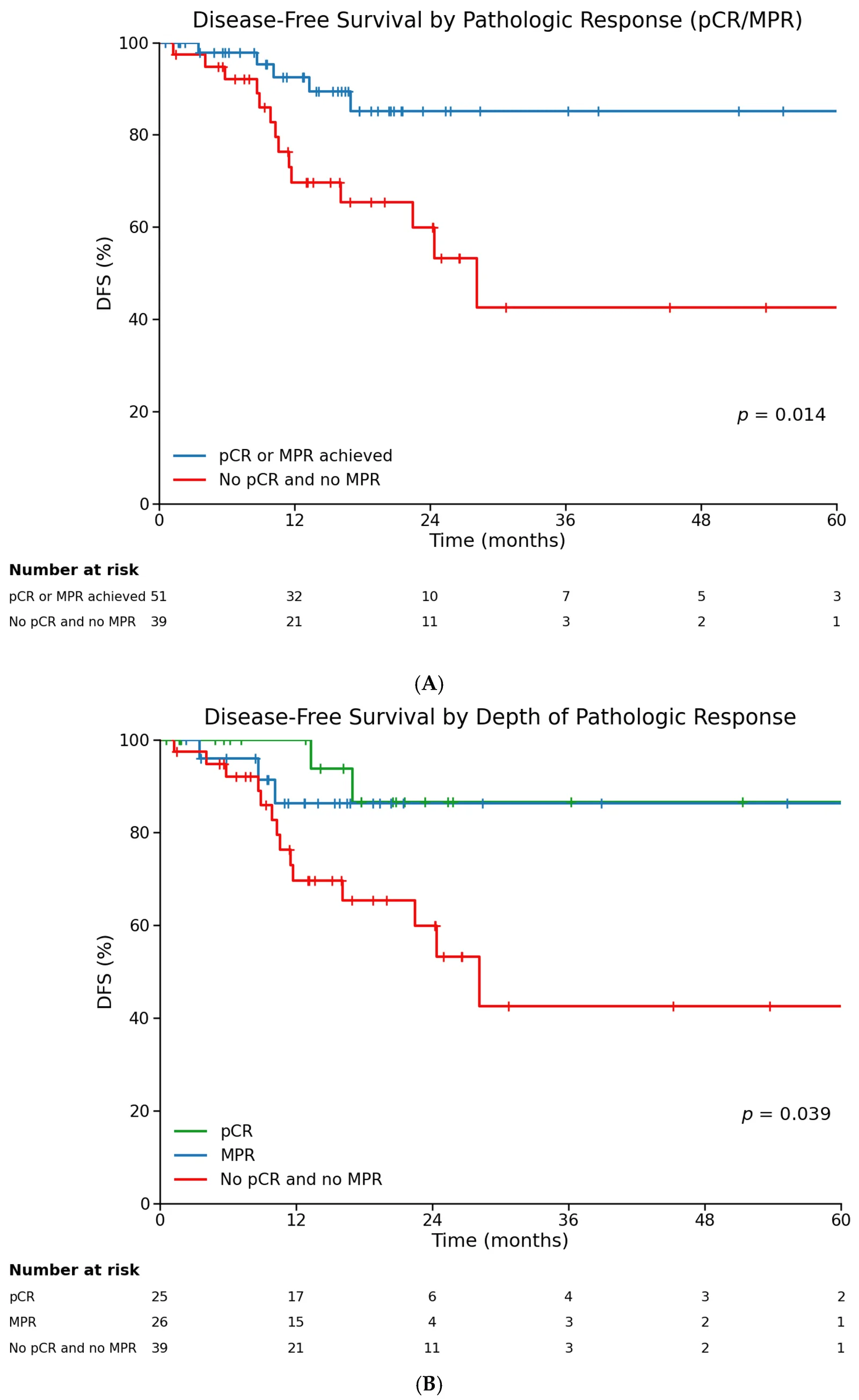
Disease-free survival according to pathologic response following neoadjuvant chemoimmunotherapy and subsequent resection. Kaplan–Meier curves representing DFS stratified by pathologic response following neoadjuvant chemoimmunotherapy and resection in patients with clinically node-positive disease (cN+). (**A**) DFS according to binary pathologic response classification: patients achieving either pathologic complete response (pCR) or major pathologic response (MPR) were classified as responders, whereas patients without pCR or MPR were classified as non-responders. Median DFS was 64.8 months (95% CI, 64.8–not reached) among responders compared to 28.2 months (95% CI, 16.1–not reached) among non-responders (log-rank *p* = 0.014). (**B**) DFS according to the depth of pathologic response: median DFS was not reached in patients achieving pCR, was 64.8 months (95% CI, not estimable) in patients with MPR, and 28.2 months (95% CI, 16.1–not reached) in patients achieving neither pCR nor MPR (global log-rank *p* = 0.039).

In total, 51 patients (56.7%) achieved pCR (*n* = 25) or MPR (*n* = 26), whereas 39 patients (43.3%) did not achieve an objective response. Pathologic response was strongly associated with DFS. Responders (pCR or MPR) had significantly better DFS than non-responders, with a median DFS of 64.8 months (95% CI, 64.8–not reached) versus 28.2 months (95% CI, 16.1–not reached), respectively (Figure 2A; log-rank *p* = 0.014). Further stratification by depth of pathologic response revealed a strong association with DFS across patients with pCR, MPR, and no objective response (Figure 2B; global log-rank *p* = 0.039). Median DFS was not reached in patients with pCR, was 64.8 months (95% CI, not estimable) in patients with MPR, and 28.2 months (95% CI, 16.1–not reached) in patients without objective response. The 64.8-month median estimates were based on sparse risk sets at late follow-up.

### 3.6. Integrated Risk Stratification Combining Pathologic Response and Nodal Status

Risk stratification integrating postoperative nodal status and pathologic response separated a clearly favorable low-risk group from a distinct high-risk group (Table 3). DFS differed significantly across these risk categories (global log-rank *p* = 0.015). Increasing risk category was associated with an increased hazard of recurrence (HR 2.14; 95% CI, 1.23–3.71; *p* = 0.007). One- and two-year DFS rates were 94.5% and 86.1% in the low-risk group; 78.8% and 78.8% in the intermediate-risk group; and 68.0% and 54.6% in the high-risk group, respectively.

### 3.7. Predictors for Residual Mediastinal Nodal Disease

In univariable analyses, higher pretreatment PD-L1 expression was associated with lower odds of residual mediastinal nodal disease, whereas baseline cN2 disease showed a numerical association with higher odds of ypN2 disease. In the restricted two-variable multivariable model, baseline cN2 disease was associated with higher odds of residual ypN2 disease compared with cN1 disease (adjusted OR 4.90, 95% CI, 1.02–23.66; *p* = 0.048). Each 10-percentage-point increase in pretreatment PD-L1 expression was associated with lower odds of residual ypN2 disease (adjusted OR 0.83, 95% CI, 0.71–0.98; *p* = 0.024). Other evaluated clinical and treatment-related variables were not significantly associated with residual mediastinal nodal disease in univariable analyses (Table 4).

### 3.8. Association Between Baseline PD-L1 Expression and Pathologic Response

Pretreatment PD-L1 expression was categorized as <1% (*n* = 10), 1–49% (*n* = 41), and ≥50% (*n* = 39). Rates of pCR differed significantly across PD-L1 expression groups (*p* = 0.013), increasing from 20.0% (2/10) in PD-L1 < 1% and 14.6% (6/41) in PD-L1 1–49% to 43.6% (17/39) in PD-L1 ≥ 50% tumors. MPR rates did not significantly differ between groups (*p* = 0.284), with rates of 30.0% (3/10), 36.6% (15/41), and 20.5% (8/39), respectively.

## 4. Discussion

Neoadjuvant chemoimmunotherapy has shifted the treatment landscape for resectable stage II–III NSCLC, with randomized phase III trials demonstrating improved event-free survival and increased pathologic response rates compared with chemotherapy alone [9,10,11]. While neoadjuvant chemoimmunotherapy yields encouraging pathologic response rates, residual nodal disease remains poorly described, and its prognostic implications in the immunotherapy era are incompletely defined. Moreover, pathologic response of the primary tumor does not necessarily translate into nodal eradication, suggesting that postoperative nodal status may independently influence outcomes [15,22,23,29].

In this retrospective cohort of patients with resectable NSCLC treated with neoadjuvant chemoimmunotherapy and surgery, postoperative pathologic nodal status was strongly associated with DFS. Residual nodal disease occurred in 41.1% of patients and was associated with significantly inferior DFS, particularly in those with residual mediastinal involvement. Recent retrospective analyses report worse progression-free and overall survival in patients with ypN+ disease compared with those achieving ypN0 after neoadjuvant immunotherapy-based regimens. Further, nodal clearance post-therapy has been shown to confer favorable long-term outcomes irrespective of baseline nodal stage, underscoring the clinical relevance of nodal assessment [15,23]. Ma et al. [23] reported significantly improved survival in patients achieving ypN0 compared with those with residual nodal disease, while Guo et al. [15] found that failure to achieve nodal downstaging after neoadjuvant chemoimmunotherapy was associated with inferior survival. A real-world analysis by Shao et al. [29] evaluated pathologic responses of the primary tumor and lymph nodes after neoadjuvant chemoimmunotherapy and frequently observed discordant responses between the primary lesion and lymphatic sites. An important knowledge gap remains, as prior investigations primarily dichotomized postoperative nodal status (ypN0 vs. ypN+) without characterizing the anatomic extent of residual nodal disease and relied on the UICC/AJCC 8th edition classification, which does not distinguish single- from multi-station mediastinal involvement. Consequently, residual N2 disease has largely been treated as a binary entity. Contemporary phase III perioperative immunotherapy trials have demonstrated improved event-free survival across stage II–III populations, yet a substantial proportion of patients experience recurrence despite surgery and systemic therapy [10,11,12]. These trials report nodal downstaging but typically analyze postoperative nodal status in aggregated categories.

To our knowledge, no previous clinical study has evaluated residual mediastinal nodal disease after neoadjuvant chemoimmunotherapy using the UICC/AJCC 9th edition nodal subclassification. Within residual N2 disease, patients with single-station (ypN2a) mediastinal involvement had longer DFS than those with multi-station (ypN2b) disease (24.4 vs. 9.9 months). However, because only six patients were classified as ypN2b, this observation should be considered exploratory and interpreted with caution. Nevertheless, the marked separation of the survival curves suggests possible prognostic heterogeneity within residual nodal disease, warranting validation in larger cohorts.

Emerging data suggest that mediastinal nodal responses are biologically distinct from those of the primary tumor. Han et al. [30] demonstrated that conventional CT-based size criteria and SUVmax reduction on PET/CT have limited accuracy for detecting residual N2 disease after neoadjuvant chemoimmunotherapy, with radiologic regression poorly correlating with pathologic nodal clearance in mediastinal stations. Our findings extend this challenge by highlighting the need for precise preoperative mediastinal nodal assessment. While nodal downstaging has long been recognized as a favorable indicator following induction chemotherapy, immunotherapy appears to alter response patterns, with substantial regression of the primary tumor sometimes occurring despite residual nodal involvement. In this context, our findings demonstrate that residual mediastinal nodal involvement remains strongly associated with recurrence risk, with markedly worse DFS in ypN2 disease despite modern neoadjuvant chemoimmunotherapy. Although the 9th edition staging system has not yet been widely adopted in prospective clinical trials, our results support its value for postoperative risk stratification.

Pathologic response was also strongly associated with DFS. Notably, the combined pCR/MPR rate in our cohort (56.7%) was comparable to, or slightly higher than, rates reported in randomized phase III neoadjuvant and perioperative chemoimmunotherapy trials (48–55%) [9,10,11,12]. Although cross-trial comparisons require cautious interpretation, this observation may reflect rigorous multidisciplinary patient selection and standardized surgical and pathological assessment within a high-volume tertiary referral center. Patients achieving pCR or MPR demonstrated improved disease control; however, MPR could coexist with residual nodal disease, and these patients experienced worse outcomes than those achieving both tumor response and nodal clearance. Integrating pathologic response and postoperative nodal status enabled robust risk stratification, separating patients into low-, intermediate-, and high-risk groups with distinct DFS patterns, consistent with Xu et al. [22]. Patients with nodal clearance and a pathologic response represented a favorable low-risk group, whereas residual mediastinal nodal disease defined a high-risk population regardless of tumor response. This combined tumor-nodal framework may provide a pragmatic basis for postoperative prognostication and adjuvant treatment strategies.

Among patients with baseline nodal involvement, greater clinical nodal burden at diagnosis was associated with an increased likelihood of residual nodal disease after neoadjuvant chemoimmunotherapy, suggesting that baseline nodal burden may influence treatment resistance at the nodal level. Baseline cN2 disease was associated with a lower probability of mediastinal nodal clearance and a higher likelihood of residual ypN2 disease after neoadjuvant chemoimmunotherapy, whereas higher pretreatment PD-L1 expression was associated with lower odds of residual ypN2 disease. Given the limited number of outcome events, these associations should be interpreted cautiously and warrant further investigation.

Several limitations of this study exist, including the retrospective, single-center design and heterogeneous treatment regimens reflecting real-world practice rather than uniform trial protocols. The cohort size and limited number of DFS events also reduce statistical power, particularly for subgroup analyses, and findings from small nodal subgroups should therefore be considered exploratory. However, all resections included standardized systematic mediastinal lymphadenectomy and were performed at a high-volume thoracic surgery center. Treatment strategies reflected the real-world implementation of evolving chemoimmunotherapy protocols during the study period. Notably, most patients were treated during the contemporary immunotherapy era, with 74 of 90 patients (82.2%) initiating treatment between 2022 and 2025. Because earlier-treated patients contributed disproportionately to long-term follow-up while treatment regimens evolved over time, a potential treatment-era effect on late survival estimates cannot be excluded. The high thoracotomy rate reflects cohort complexity, as 35.6% of patients underwent major extended or reconstructive resections, including pneumonectomy, sleeve resection, bilobectomy, or carinal resection, wherein previous studies have similarly reported increased surgical complexity, conversion to thoracotomy, and challenging dissections after neoadjuvant immunotherapy due to treatment-associated fibrosis and inflammatory nodal changes [31,32,33]. In addition, postoperative therapy was largely protocol-driven and determined before treatment rather than by postoperative pathologic findings. The relatively short follow-up and limited number of DFS events limit the precision of long-term survival estimates, particularly in smaller subgroups, and estimates at later time points should therefore be interpreted with caution. However, the consistent separation of survival curves by postoperative nodal status supports the biological and clinical relevance of these findings. The nodal yield observed in this cohort is consistent with guideline-concordant systematic dissection in the post-neoadjuvant setting and supports the adequacy of postoperative pathologic staging [34,35,36,37]. Nevertheless, this analysis provides clinically relevant insights into the prognostic implications of residual nodal disease after neoadjuvant chemoimmunotherapy. Using the UICC/AJCC 9th edition nodal subclassification, our findings suggest that residual mediastinal nodal disease may not represent a uniform entity and that its anatomic extent (single- vs. multi-station) may help identify clinically relevant prognostic heterogeneity. Further validation in larger multicenter datasets is warranted. These results support the clinical relevance of refined mediastinal nodal classification for postoperative risk stratification in the immunotherapy era.

## 5. Conclusions

Residual nodal disease after neoadjuvant chemoimmunotherapy and curative surgery was associated with progressively inferior disease-free survival with increasing postoperative nodal burden and remained prognostically relevant independently of pathologic response of the primary tumor. The UICC/AJCC 9th-edition nodal subclassification provided additional prognostic granularity by capturing clinically relevant differences in the anatomic extent of residual nodal disease. These findings underscore postoperative nodal burden as a key determinant of outcome and support precise mediastinal reassessment after neoadjuvant chemoimmunotherapy and further investigation of adjuvant treatment strategies informed by residual nodal disease.

## Figures and Tables

**Table 1:** Baseline characteristics of the study cohort.

Characteristics	*n* (%)
Sex	
Male	54 (60.0)
Female	36 (40.0)
Age at diagnosis, years	
Median (IQR)	63 (58–69)
Histology	
Adenocarcinoma	52 (57.8)
Squamous cell carcinoma	38 (42.2)
Clinical stage (UICC/AJCC 8th edition)	
IIB	8 (8.9)
IIIA	32 (35.6)
IIIB	50 (55.6)
Tumor location—side	
Right lung	50 (55.6)
Left lung	38 (42.2)
Central/carinal	2 (2.2)
Anatomic locations	
Right upper lobe	36 (40.0)
Middle lobe	1 (1.1)
Lower lobe	9 (10.0)
Right central/hilar (>1 lobe)	4 (4.4)
Left upper lobe	27 (30.0)
Left lower lobe	7 (7.8)
Left central/hilar (>1 lobe)	4 (4.4)
Carinal tumors	2 (2.2)
Time from end of neoadjuvant therapy to surgery, weeks, median (IQR)	5.6 (4.0–6.7)
Surgical approach	
Open thoracotomy	72 (80.0)
VATS	18 (20.0)
Extent of resection—overall	
Lobectomy	69 (76.7)
Bilobectomy	6 (6.7)
Pneumonectomy	13 (14.4)
Carinal resection	2 (2.2)
Extent of resection by side	
Lobectomy—right	40 (44.4)
Lobectomy—left	29 (32.2)
Bilobectomy—right	6 (6.7)
Pneumonectomy—right	4 (4.4)
Pneumonectomy—left	9 (10.0)
Carinal resection—central	2 (2.2)
Sleeve lobar resections	
Any sleeve lobar resection	11 (12.2)
Right upper lobe sleeve	5 (5.6)
Right lower lobe sleeve	1 (1.1)
Left upper lobe sleeve	4 (4.4)
Left lower lobe sleeve	1 (1.1)

**Table 2:** Residual pathologic nodal disease according to the 8th and 9th editions of the UICC/AJCC TNM staging systems.

8th Edition	9th Edition	Definition	*n*	%
ypN0	ypN0	Nodal clearance	53	58.9
ypN1	ypN1	Ipsilateral peribronchial and/or ipsilateral hilar nodal involvement	16	17.8
ypN2		Residual mediastinal nodal involvement	21	23.3
	ypN2a	Single-station mediastinal disease	15	16.7
	ypN2b	Multi-station mediastinal disease	6	6.7
Any ypN+		ypN1 or ypN2 disease	37	41.1

**Table 3:** Integrated risk stratification combining pathologic response and residual nodal disease.

Postoperative Nodal Status	pCR	MPR	No Objective Response	Total
ypN0	Low (*n* = 25)	Low (*n* = 18)	Intermediate (*n* = 10)	53
ypN1	Intermediate (*n* = 0)	Intermediate (*n* = 4)	High (*n* = 12)	16
ypN2	High (*n* = 0)	High (*n* = 4)	High (*n* = 17)	21
Total	25	26	39	90

**Table 4:** Logistic regression analysis of factors associated with residual mediastinal nodal disease.

Predictor	Unadjusted OR (95% CI)	*p*-Value	Adjusted OR (95% CI)	*p*-Value
Age, per 10-year increase	1.57 (0.83–2.97)	0.161	—	—
Male sex	1.11 (0.41–3.03)	0.839	—	—
Histology, squamous cell carcinoma vs. adenocarcinoma	0.80 (0.29–2.18)	0.662	—	—
Pack-years, per 10-pack-year increase	1.02 (0.82–1.27)	0.865	—	—
Clinical T3–4 vs. T1–2	0.88 (0.30–2.62)	0.822	—	—
Clinical N2 vs. N1	4.45 (0.95–20.79)	0.058	4.90 (1.02–23.66)	0.048
Pretreatment PD-L1, per 10-percentage-point increase	0.84 (0.72–0.98)	0.028	0.83 (0.71–0.98)	0.024
Neoadjuvant treatment cycles, per cycle	0.89 (0.49–1.61)	0.692	—	—
Time from treatment completion to surgery, per week	1.10 (0.90–1.34)	0.363	—	—

Footnote: OR, odds ratio; CI, confidence interval; PD-L1, programmed death-ligand 1. The outcome was residual mediastinal nodal disease, defined as ypN2 versus ypN0/ypN1. Univariable models were fitted separately for each candidate predictor. Because only 21 patients had residual ypN2 disease, the multivariable model was deliberately restricted to baseline clinical nodal status and pretreatment PD-L1 expression to reduce overfitting and preserve model stability. Dashes indicate variables evaluated only in univariable analyses and not entered into the restricted multivariable model.

## Data Availability

The data supporting the findings of this study are available from the corresponding author upon reasonable request.

## Abbreviations

The following abbreviations are used in this manuscript:

AJCC	American Joint Committee on Cancer
CI	Confidence interval
DFS	Disease-free survival
EBUS-TBNA	Endobronchial ultrasound-guided transbronchial needle aspiration
HR	Hazard ratio
IQR	Interquartile range
LNR	Lymph node ratio
MPR	Major pathologic response
NSCLC	Non-small cell lung cancer
OR	Odds ratio
pCR	Pathologic complete response
PD-L1	Programmed death-ligand 1
PET/CT	Positron emission tomography/computed tomography
UICC	Union for International Cancer Control
VATS	Video-assisted thoracoscopic surgery

## References

[1] Remon J., Soria J.C., Peters S., ESMO Guidelines Committee (2021). Early and locally advanced non-small-cell lung cancer: An update of the ESMO Clinical Practice Guidelines focusing on diagnosis, staging, systemic and local therapy. Ann. Oncol..

[2] Rajaram R., Huang Q., Li R.Z., Chandran U., Zhang Y., Amos T.B., Wright G.W., Ferko N.C., Kalsekar I. (2024). Recurrence-Free Survival in Patients With Surgically Resected Non-Small Cell Lung Cancer: A Systematic Literature Review and Meta-Analysis. Chest.

[3] Gilligan D., Nicolson M., Smith I., Groen H., Dalesio O., Goldstraw P., Hatton M., Hopwood P., Manegold C., Schramel F. (2007). Preoperative chemotherapy in patients with resectable non-small cell lung cancer: Results of the MRC LU22/NVALT 2/EORTC 08012 multicentre randomised trial and update of systematic review. Lancet.

[4] Burdett S., Stewart L.A., Rydzewska L. (2006). A systematic review and meta-analysis of the literature: Chemotherapy and surgery versus surgery alone in non-small cell lung cancer. J. Thorac. Oncol..

[5] Burdett S.S., Stewart L.A., Rydzewska L. (2007). Chemotherapy and surgery versus surgery alone in non-small cell lung cancer. Cochrane Database Syst. Rev..

[6] Pirker R. (2014). Adjuvant chemotherapy in patients with completely resected non-small cell lung cancer. Transl. Lung Cancer Res..

[7] Shukla N., Hanna N. (2021). Neoadjuvant and Adjuvant Immunotherapy in Early-Stage Non-Small Cell Lung Cancer. Lung Cancer.

[8] Zhang W., Liang Z., Zhao Y., Li Y., Chen T., Li W., Chen Y., Wu P., Zhang H., Fang C. (2024). Efficacy and safety of neoadjuvant immunotherapy plus chemotherapy followed by adjuvant immunotherapy in resectable non-small cell lung cancer: A meta-analysis of phase 3 clinical trials. Front. Immunol..

[9] Forde P.M., Spicer J., Lu S., Provencio M., Mitsudomi T., Awad M.M., Felip E., Broderick S.R., Brahmer J.R., Swanson S.J. (2022). Neoadjuvant Nivolumab plus Chemotherapy in Resectable Lung Cancer. N. Engl. J. Med..

[10] Wakelee H., Liberman M., Kato T., Tsuboi M., Lee S.-H., Gao S., Chen K.-N., Dooms C., Majem M., Eigendorff E. (2023). Perioperative Pembrolizumab for Early-Stage Non-Small-Cell Lung Cancer. N. Engl. J. Med..

[11] Heymach J.V., Harpole D., Mitsudomi T., Taube J.M., Galffy G., Hochmair M., Winder T., Zukov R., Garbaos G., Gao S. (2023). Perioperative Durvalumab for Resectable Non-Small-Cell Lung Cancer. N. Engl. J. Med..

[12] Cascone T., Awad M.M., Spicer J.D., He J., Lu S., Sepesi B., Tanaka F., Taube J.M., Cornelissen R., Havel L. (2024). Perioperative Nivolumab in Resectable Lung Cancer. N. Engl. J. Med..

[13] Provencio M., Serna-Blasco R., Nadal E., Insa A., García-Campelo M.R., Rubio J.C., Dómine M., Majem M., Rodríguez-Abreu D., Martínez-Martí A. (2022). Overall Survival and Biomarker Analysis of Neoadjuvant Nivolumab Plus Chemotherapy in Operable Stage IIIA Non-Small-Cell Lung Cancer (NADIM phase II trial). J. Clin. Oncol..

[14] Forde P.M., Spicer J.D., Provencio M., Mitsudomi T., Awad M.M., Wang C., Lu S., Felip E., Swanson S.J., Brahmer J.R. (2025). Overall Survival with Neoadjuvant Nivolumab plus Chemotherapy in Lung Cancer. N. Engl. J. Med..

[15] Guo M., Abdel-Rasoul M., Chang J., Guo A., Baiu I., D’Souza D., Merritt R., Kneuertz P. (2025). The Prognostic Value of Lymph Node Downstaging Following Neoadjuvant Chemoimmunotherapy for Non-Small Cell Lung Cancer. J. Cancer.

[16] Gautschi O., Minervini F., Kestenholz P. (2020). Persistent N2 disease after neoadjuvant treatment...and now?—The oncologist view. Curr. Chall. Thorac. Surg..

[17] Muñiz S.M., Sánchez S.M., Rodríguez J.C., Vila B.L., Herrero E.L., Díaz M.D.H., González V.V.B., Valladares B.T., Barrón B.V., Jimenez F.J.M. (2021). Advances in multimodal treatment for stage IIIA-N2 non-small cell lung cancer. J. Clin. Transl. Res..

[18] Isgorucu O., Citak N. (2023). Survival Analysis of Surgically Resected ypN2 Lung Cancer after Neoadjuvant Therapy. Thorac. Cardiovasc. Surg..

[19] Chen Y., Peng X., Zhou Y., Xia K., Zhuang W. (2018). Comparing the benefits of chemoradiotherapy and chemotherapy for resectable stage III A/N2 non-small cell lung cancer: A meta-analysis. World J. Surg. Oncol..

[20] Tong S., Qin Z., Wan M., Zhang L., Cui Y., Yao Y. (2018). Induction chemoradiotherapy versus induction chemotherapy for potentially resectable stage IIIA (N2) non-small cell lung cancer: A systematic review and meta-analysis. J. Thorac. Dis..

[21] Xu Y.P., Li B., Xu X.L., Mao W.M. (2015). Is There a Survival Benefit in Patients With Stage IIIA (N2) Non-small Cell Lung Cancer Receiving Neoadjuvant Chemotherapy and/or Radiotherapy Prior to Surgical Resection: A Systematic Review and Meta-analysis. Medicine.

[22] Xu Y., Ma D., Qin Y., Liu H. (2025). Prognostic significance of pathological response and lymph node status in neoadjuvant immunotherapy for potentially resectable non-small cell lung cancer. Ann. Med..

[23] Ma R., Yang H., Ge Y., Ma T., Wang J., Li S., Feng T., Feng S., Zhang C., Sun T. (2025). Prognostic Implications of Lymph Node Status in Non-Small-Cell Lung Cancer Patients Before and After Neoadjuvant Chemoimmunotherapy: A Multicenter Retrospective Study. Clin. Lung Cancer..

[24] Rami-Porta R., Nishimura K.K., Giroux D.J., Detterbeck F., Cardillo G., Edwards J.G., Fong K.M., Giuliani M., Huang J., Kernstine K.H. (2024). The International Association for the Study of Lung Cancer Lung Cancer Staging Project: Proposals for Revision of the TNM Stage Groups in the Forthcoming (Ninth) Edition of the TNM Classification for Lung Cancer. J. Thorac. Oncol..

[25] Spicer J.D., Cascone T., Wynes M.W., Ahn M.-J., Dacic S., Felip E., Forde P.M., Higgins K.A., Kris M.G., Mitsudomi T. (2024). Neoadjuvant and Adjuvant Treatments for Early Stage Resectable NSCLC: Consensus Recommendations From the International Association for the Study of Lung Cancer. J. Thorac. Oncol..

[26] Aigner C., Baldes N., Begic M., Doerr F., Hoda M.A., Bölükbas S. (2025). Current perspective on resectability in stage III locally advanced NSCLC—The thoracic surgeons’ view. Eur. J. Cancer..

[27] Houda I., Bahce I., Dickhoff C., Kroese T.E., Kroeze S.G., Mariolo A.V., Tagliamento M., Moliner L., Brandão M., Pretzenbacher Y. (2025). An international and multidisciplinary EORTC survey on resectability of stage III non-small cell lung cancer. Lung Cancer.

[28] Travis W.D., Dacic S., Wistuba I., Sholl L., Adusumilli P., Bubendorf L., Bunn P., Cascone T., Chaft J., Chen G. (2020). IASLC Multidisciplinary Recommendations for Pathologic Assessment of Lung Cancer Resection Specimens After Neoadjuvant Therapy. J. Thorac. Oncol..

[29] Shao W., Zhao C., Li H., Sun J., Li B., Liu Z., Chen F., Liu J., Guo X., Guo H. (2024). Pathological responses of primary tumor and lymph nodes in non-small cell lung cancer after neoadjuvant chemoimmunotherapy: A retrospective real-world study. J. Thorac. Dis..

[30] Han T., Cheng S., Wang X., Qi Q., Chen J., Wang W., Zhou J., Li Y., Chen K., Li H. (2025). Prognostic Significance, Radiological, and Metabolic Characteristics of Metastatic Lymph Nodes in Resectable Non-Small Cell Lung Cancer Following Neoadjuvant Chemoimmunotherapy. Thorac. Cancer..

[31] Stefani D., Plönes T., Viehof J., Darwiche K., Stuschke M., Schuler M., Aigner C. (2021). Lung Cancer Surgery after Neoadjuvant Immunotherapy. Cancers.

[32] Minervini F., Kestenholz P.B., Kocher G.J., Azenha F. (2023). Thoracic surgical challenges after neoadjuvant immunotherapy for non-small cell lung cancer: A narrative review. AME Surg. J..

[33] Sepesi B., Zhou N., William W.N., Lin H.Y., Leung C.H., Weissferdt A., Mitchell K.G., Pataer A., Walsh G.L., Rice D.C. (2022). Surgical outcomes after neoadjuvant nivolumab or nivolumab with ipilimumab in patients with non-small cell lung cancer. J. Thorac. Cardiovasc. Surg..

[34] Lardinois D., De Leyn P., Van Schil P., Porta R.R., Waller D., Passlick B., Zielinski M., Junker K., Rendina E., Ris H. (2006). ESTS guidelines for intraoperative lymph node staging in non-small cell lung cancer. Eur. J. Cardiothorac. Surg..

[35] Rami-Porta R., Wittekind C., Goldstraw P., International Association for the Study of Lung Cancer (IASLC) Staging Committee (2005). Complete resection in lung cancer surgery: Proposed definition. Lung Cancer.

[36] Rusch V.W., Asamura H., Watanabe H., Giroux D.J., Rami-Porta R., Goldstraw P. (2009). The IASLC lung cancer staging project: A proposal for a new international lymph node map in the forthcoming seventh edition of the TNM classification for lung cancer. J. Thorac. Oncol..

[37] Zer A., Ahn M.-J., Barlesi F., Bubendorf L., De Ruysscher D., Garrido P., Gautschi O., Hendriks L., Jänne P., Kerr K. (2025). Early and locally advanced non-small-cell lung cancer: ESMO Clinical Practice Guideline for diagnosis, treatment and follow-up. Ann. Oncol..

